# Resiquimod and polyinosinic–polycytidylic acid formulation with aluminum hydroxide as an adjuvant for foot-and-mouth disease vaccine

**DOI:** 10.1186/1746-6148-10-2

**Published:** 2014-01-03

**Authors:** Chun-Xue Zhou, Dong Li, Ying-Li Chen, Zeng-Jun Lu, Pu Sun, Yi-Mei Cao, Hui-Fang Bao, Yuan-Fang Fu, Ping-Hua Li, Xing-Wen Bai, Bao-Xia Xie, Zai-Xin Liu

**Affiliations:** 1 State Key Laboratory of Veterinary Etiological Biology, Key Laboratory of Animal Virology of Ministry of Agriculture, OIE/National Foot-and-Mouth Disease Laboratory, Lanzhou Veterinary Research Institute, Chinese Academy of Agricultural Sciences, Lanzhou 730046, China

**Keywords:** R848, Poly(I:C), Aluminum hydroxide, FMDV

## Abstract

**Background:**

Toll-like receptor (TLR) agonists reportedly have potent antiviral and antitumor activities and may be a new kind of adjuvant for enhancing immune efficacy. Resiquimod (R848) is an imidazoquinoline compound with potent antiviral activity and functions through the TLR7/TLR8 MyD88-dependent signaling pathway. Polyinosinic-polycytidylic acid [poly(I:C)] is a synthetic analog of double-stranded RNA that induces the production of pro-inflammatory cytokines by the activation of NF-κB through TLR3. This study investigated the potential of R848 and poly(I:C) as an adjuvant 146S foot-and-mouth disease virus (FMDV) vaccine formulated with aluminum hydroxide (Al(OH)_3_).

**Results:**

Antibody titers to FMDV and CD8^+^ T cells were markedly enhanced in mice immunized to 146S FMDV + Al(OH)_3_ + R848 + poly(I:C) compared with mice immunized to FMDV + ISA206. IFN-γ secretion substantially increased compared with IL-4 secretion by splenic T cells stimulated with FMDV antigens in vitro, suggesting that R848, poly(I:C), and with Al(OH)_3_ together biased the immune response toward a Th1-type direction.

**Conclusions:**

These results indicated that the R848 and poly(I:C) together with Al(OH)_3_ enhanced humoral and cellular immune responses to immunization with 146S FMDV antigens. Thus, this new vaccine formulation can be used for FMDV prevention.

## Background

Foot-and-mouth disease virus (FMDV), which belongs to family *Picornaviridae*, genus *Aphthovirus*, causes a highly contagious and devastating disease in cloven-hoofed animals, such as cattle, swine, sheep, and goats [1]. FMDV was first discovered by Loeffler and Frosch in the late nineteenth century [2]. Since then, outbreaks of foot-and-mouth disease (FMD) in Asia and Europe have led to the slaughter of millions of infected animals and billions of dollar loss [3,4].

FMD, which has been reported in many areas of the world, has the features of fever, short-lived viremia, and lesions on feet and tongue. The disease can be controlled by vaccination of chemically inactivated whole virus formulated with adjuvant. Aluminum salts are the only ones licensed in human vaccines and mainly enhances Th2-specific immune responses. Extensive clinical data have demonstrated that aluminum salt vaccines have excellent safety profiles and limited adverse reactions in injection sites. Both aluminum hydroxide (Al(OH)_3_) adjuvant and mineral oil adjuvant have been applied to control FMD. However, the antibody titers were higher and the morbidity rates were lower in cattle, sheep, and pigs when emulsified oil is used as an adjuvant [5]. Moreover, the ability of the aluminum adjuvant to enhance Th1 cell-mediate immune responses is insufficient. However, mineral oil adjuvant can induce local and general reactions, such as granuloma, gangrene, or fever [6].

Toll-like receptors (TLRs) are pattern recognition receptors that play an important role in innate immune response. TLRs are preferentially expressed in lymphocytes, dendritic cells (DCs), and macrophages [7]. The engagement of TLRs in DCs links innate immunity and adaptive immunity, which is vital for immune cell maturation, cell differentiation (Th1/Th2) based on pro-inflammatory cytokines, and proliferation of antigen-specific CD4^+^ and CD8^+^T cells [8]. To date, 13 TLRs have been confirmed in both humans and mice. Many natural or synthetic ligands that recognize the TLRs have also been discovered [9]. TLR7, which favors a TH1 response, can be activated by some synthetic agonists, such as imiquimod [10,11], resiquimod (R848) [12,13], and 3 M-052 [14]. Imiquimod and R848 have been demonstrated to have immune response properties in terms of antiviral and antitumor activities. Imiquimod, the first synthetic imidazoquinoline, is approved for the topical treatment of genital warts, skin cancer, superficial basal cell carcinoma, and actinic keratosis [15]. Through conventional subcutaneous and intramuscular vaccination, R848 shows that these molecules could enhance DCs maturation, as well as cellular and humoral adaptive immunities in mice, rats, guinea pigs, and monkeys [16].

Polvriboinsine–polyribocyaidylic acid [poly(I:C)] is a synthetic analog of double-stranded RNA that could recognize TLR3 and lead to the activation of NF-κB and the secretion of pro-inflammatory cytokines. Poly(I:C) has been shown to induce the production of IL-12 and IFN-γ, which could shift the Th2-type response to Th1-type response.

Thus far, ISA206 is considered the most effective oil adjuvant in FMD vaccines for protecting animals against infection. To determine if other types of adjuvants are more effective than ISA206, this study was carried out to determine the ability of R848 and poly(I:C) in assisting Al(OH)_3_ as FMD vaccine.

## Methods

### Reagents and virus

R848 and poly(I:C) were manufactured by Sigma–Aldrich (St. Louis, MO, USA). ISA206 was purchased from Seppic Company (France). Al(OH)_3_ (pH 7.4) was prepared in our laboratory and stored at 4°C.

The FMDV strain used was a type-O FMDV vaccine strain isolated and stored in our laboratory. The procedures for virus preparation were performed as previously described [17]. This strain was propagated in a BHK-21 cell line and inactivated using binary ethyleneimine for 24 h at 37°C. Then, 146S antigen was concentrated and purified by sucrose density gradient centrifugation. In a typical procedure, appropriately inactivated virus samples were centrifuged through a cushion of 20% sucrose in TNE buffer [containing 10 mM Tris–HCl (pH 7.5), 0.1 M NaCl, and 1 mM EDTA] in an AH-625 rotor (Sorvall) at 25 000 rpm, 2.5 h, and 4°C. The viral pellet was resuspended in TNE and centrifuged in 25%-45% (w/w) sucrose density gradients in the same buffer at 37 000 rpm, 1 h, and 4°C in a SW40 rotor (Beckman). After centrifugation, the gradients were scanned at 254 nm using UA-5 or UA-6 absorbance detectors. To prepare the whole virus particle standard, sucrose was removed by ultrafiltration using Centricon Plus-70 filter devices with Amicon Ultracel PL-30 membranes (Millipore, USA) following instructions from the manufacturer.

### Animals

Female BALB/c mice (purchased from Lanzhou University, China), approximately 5 weeks to 8 weeks old, were used in this study. They were acclimated for a week before studies begun, i.e., maintained in standard mouse chow and conditions in an animal facility. All mice were weighed prior to initiation to provide a baseline for detecting the systemic toxicity of treatments that could cause weight loss. The State Key Laboratory of Veterinary Etiological Biology and Key Laboratory of Animal Virology of the Ministry of Agriculture approved all protocols using animals (permission number: CNAS-BL0012.date:2011.11-2016.11).

### Immunization protocols

Mice in six groups (twelve mice each) were intramuscularly (IM) injected once in posterior limbs with one of the following formulations: (1) 146S + ISA206 (F + 206), (2) 146S + Al(OH)_3_ (F + A), (3) 146S + Al(OH)_3_ + 20 μg poly(I:C) (F + A + P), (4) 146S + Al(OH)_3_ + 20 μg R848 (F + A + R), (5) 146S + Al(OH)_3_ + 20 μg poly(I:C) + 20 μg R848 (F + A + P + R), and (6) placebo (PBS). 146S FMDV was administrated at 2.0 μg per mouse. All tested mice were bled before immunization (day 0) and then immunized. Blood samples were obtained at 3, 7, 14, 21, 28, and 35 days post-vaccination (dpv). Sera were separated by centrifugation at 4000 rpm for 10 min and stored at-20°C until antibody titers were tested.

### Determination of FMDV type O specific antibody titers

To measure FMDV type O-specific antibody titers following immunization, blood was obtained from each mouse. ELISA was performed according to the specifications in an LPB-ELISA commercial kit (LVRI, China). In a typical procedure, the ELISA plates were coated with rabbit antiserum against FMDV type O overnight at room temperature. Serum sample dilutions (from 1:4 to 1:512) were incubated overnight at 4°C at a pre-titrated dose of the corresponding virus strain (FMDV O type in this study) in a saline buffer liquid phase. The next day, the mixtures in the serum dilution plate were transferred into the ELISA plate and incubated for 1 h at 37°C. After a washing step (total of five times with PBST), a second incubation was carried out with guinea pig antiserum against FMDV O type for 1 h at 37°C. After a wash step similar to the one above, 50 μL of prepared rabbit anti-Guinea IgG/HRP was added to each well. Well were then covered with a plate sealer and incubated for 1 h at 37°C. Then, 50 μL of the substrate/chromophore mixture (H_2_O_2_/OPD) was added to each well, and the plate (without plate sealer) was incubated for 15 min at room temperature in the dark. Finally, 50 μL of stop solution was added to each well, and absorbance at 492 nm was read within 15 min of stopping the reaction.

### Preparation of splenic lymphocytes

Individual spleens were cut into small pieces with scissors. Single cell suspension was prepared by gently squeezing through a 70 μm cell strainer. Leukocytes were isolated by density gradient centrifugation for 20 min at 1000 × *g* using Ez-Sep Mouse 1 × lymphocyte separation medium (Dakewe, China). The portion of the medium containing lymphocyte was transferred into a new tube and then washed with RPMI 1640. Cells were isolated by density gradient centrifugation for 10 min at 450 × *g*, and the supernatant was discarded. Finally, cells were resuspended in RPMI 1640 (containing penicillin/streptomycin) supplemented with 5% FCS at 5 × 10^6^ cells/mL and stored at 4°C.

### Detection of IFN-γ/IL-4

About 5 × 10^5^ spleen lymphocytes were added to each well of a 96-well microtiter plate at a final volume of 100 μL. Cells from each spleen or pools of spleens were added to each of 9 wells, 3 for PBS control, 3 for PHA control (10 μg/mL; Sigma), and 3 for 2 μg/ml of specific antigen (146S FMDV) challenge. The cells were then incubated for 48 h at 37°C in a humidified atmosphere of 5.0% CO_2_ in air. The plates were then centrifuged for 10 min at 4000 rpm to settle cells to the well bottom, and the medium was removed for analysis of IFN-γ and IL-4 production by ELISA (BD Company, USA).

### Detection of CD3^+^CD4^+^T and CD3^+^CD8^+^T cells

For CD3^+^CD4^+^ and CD3^+^CD8^+^T cell staining, total spleen lymphocytes from immunized mice were isolated and stained with anti-CD3-ALEXA FLUOR®488 & anti-CD4-ALEXA FLUOR®647 or anti-CD3-ALEXA FLUOR®488 & anti-CD8-ALEXA FLUOR®647(BD Phamingen, USA) in darkness for 20 min. Cells were isolated by density gradient centrifugation for 10 min at 3000 rpm. After discarding the supernatant, cells were twice washed with PBS and resuspended in 0.5 mL of PBS. The cells were then analyzed with a FACSAria (BD) within 4 h.

### Statistical analyses

The statistical significance of the differences in the means of experimental groups was determined by one- or two-way ANOVA analysis. Results are expressed as the mean ± standard error of mean. A difference was deemed statistically significant if *p* < 0.05.

## Results

### Effects of R848 and poly(I:C) on naïve splenocytes in vitro

**Figure 1:**
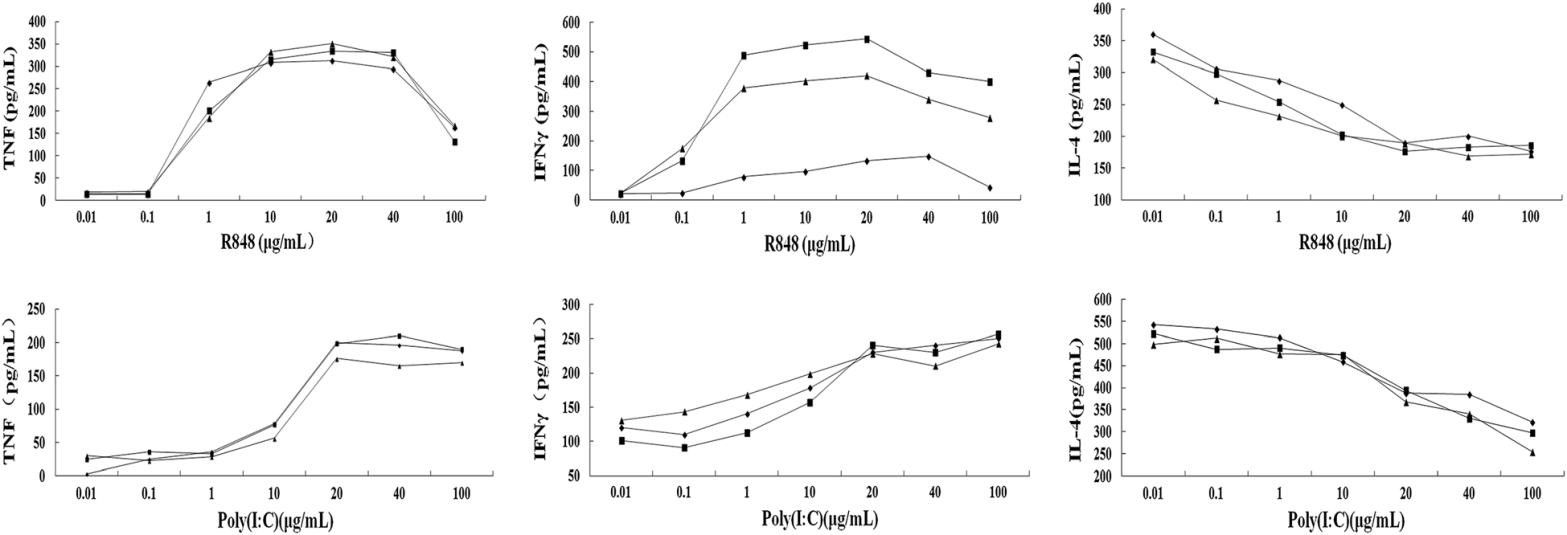
**In vitro immune activation of BALB/c mice splenocytes by R848 and poly(I:C).** Naїve BALB/c mice splenocytes (10^6^/ml) were incubated with 0.01, 0.1, 1, 10, 20, 40, and 100 μg/ml R-848 or 0.01, 0.1, 1, 10, 20, 40, and 100 μg/ml poly(I:C). Culture supernatants were obtained 48 h after stimulation and assayed for TNFα, IFN-γ, and IL-4 using ELISA. The results shown are representatives of three independent experiments.

Naïve BALB/c splenocytes were prepared and stimulated with either R848 (0.01, 0.1, 1, 10, 20, 40, 100 μg/mL) or poly(I:C) (0.01, 0.1, 1, 10, 20, 40, and 100 μg/mL). ConA (Sigma Chemical Company, USA) were used as positive control, and PBS was used as negative control (Figure 1). Splenocytes were cultured for 48 h, and cytokine induction was measured by harvesting splenocyte supernatants. To investigate the effect of R848 and poly(I:C) on the changes in Th1 and Th2 immune response in vitro, splenocyte supernatants were measured with a commercially available kit (BD company, USA). When tested for the ability to promote the induction of several different cytokines, both R848 and poly(I:C) induced the highest levels of TNF and IFNγ (Th1 cytokine) at 20 μg/mL. R848 appeared to be superior to poly(I:C) in inducing TNF and IFNγ. However, R848 and poly(I:C) induced the lowest levels of IL-4 (Th2 cytokine) when they were administered at 20 μg/mL. Results revealed that both R848 and poly(I:C) regulated the production of selective Th1 or Th2 cytokines, which favor a Th1 bias [18].

### Antibody response

**Figure 2:**
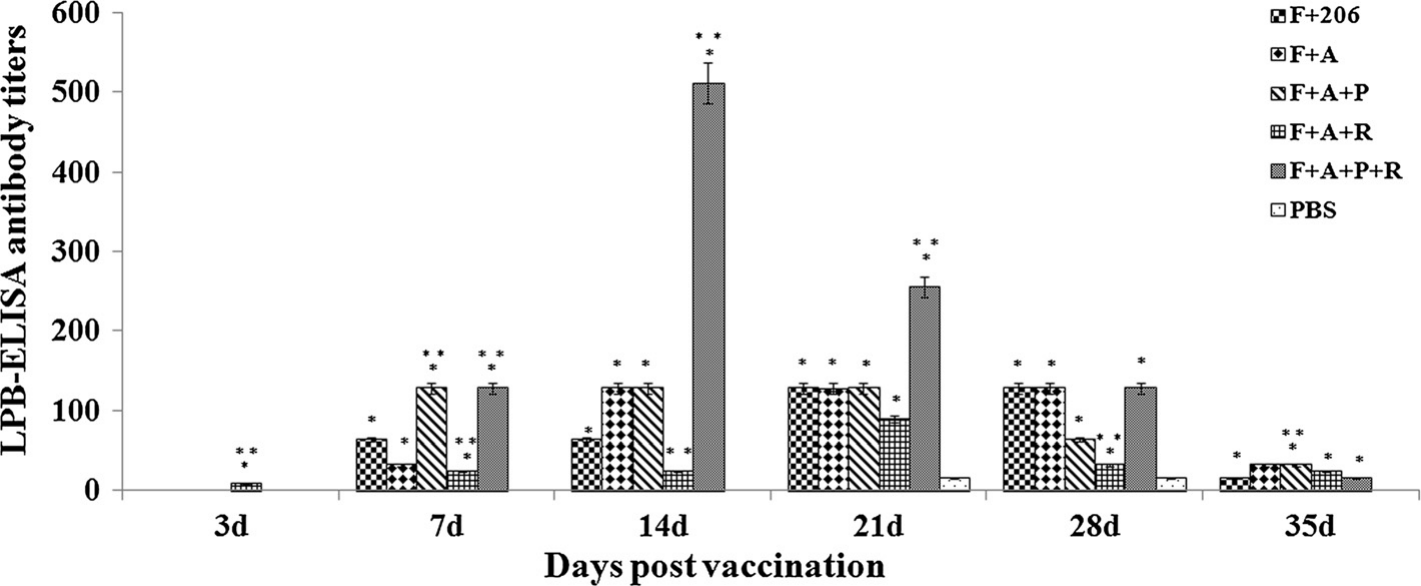
**Enhancement of anti-FMDV IgG antibody by the immunization of FMDV antigens together with R848 and poly(I:C).** Mice were bled at different dpv’s. Sera from three mice were pooled together within the same group, and total IgG anti-FMDV antibody titers were detected using ELISA. One asterisk indicates that *p* < 0.05 when comparing results from the ISA206, F + A, F + A + P, F + A + R, or F + A + P + R groups to the PBS group. Two asterisks indicate *p* < 0.05 when comparing results from the ISA206, F + A + P, F + A + R, or F + A + P + R groups with the F + A group. Each bar represents the mean antibody titer ± standard error of 12 mice per group.

Humoral immune responses were analyzed by screening for serum IgG using liquid-blocking ELISA specific for FMDV type O. Sera were collected prior to immunization and at different days after the immunization. The levels of anti-FMDV type O IgG at different days following immunization to FMDV formulated with different materials are shown in Figure 2. Pre-immune sera (day 0) were detected and found negative for anti-FMDV antibodies. Immunization to FMDV formulated with alum and R848 induced a weaker IgG antibody response than the F + A group. When mice were immunized with FMDV formulated with alum and poly(I:C), the obtained antibody titers were higher and almost equivalent to that in the F + A group. However, immunization to FMDV formulated with alum, R848, and poly(I:C) induced a striking, fourfold higher antibody, even higher than that in the F + 206 group at 14 and 21 dpv (*p* < 0.05). All above results showed that the combination of R848 and poly(I:C) played an important role in boosting anti-FMDV antibodies.

### Stimulation of immunized mice splenocytes in vitro

**Figure 3:**
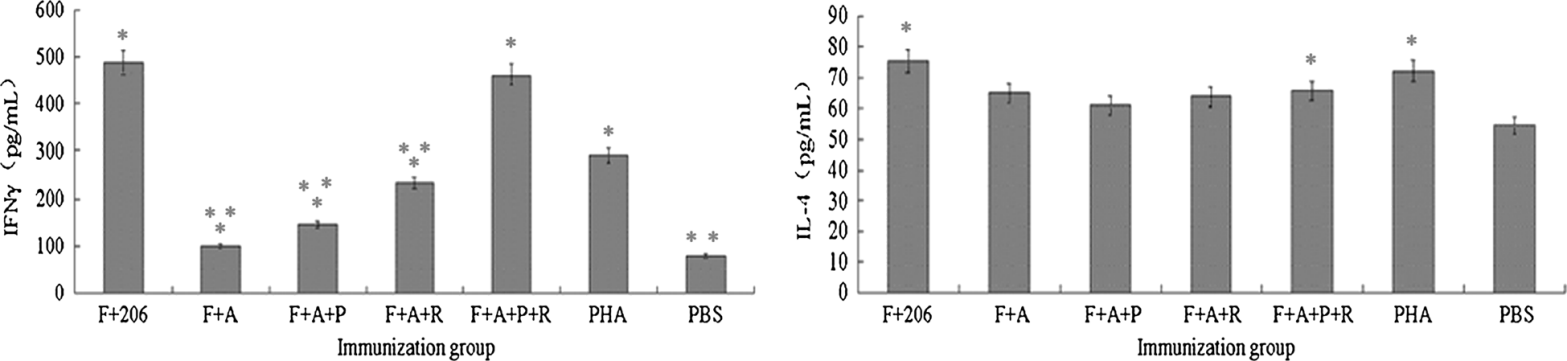
**Induction of IFN-γ and IL-4 by the immunized mice splenocytes.** Mice were sacrificed at 35 dpv. Spleens were isolated and ground into single cells under sterile conditions. Single spleen lymphocyte suspensions (5 × 10^6^ /ml) were obtained using commercial EZ-SepTM Mouse 1× Lymphocyte Separation Medium. About 200 μl of cells was stimulated by FMDV antigens as specific antigen, PHA as positive control, and PBS as negative control .One asterisk indicates that *p* < 0.05 when comparing results from the ISA206, F + A, F + A + P, F + A + R, F + A + P + R, or PHA groups to the PBS group. Two asterisks indicate *p* < 0.05 when comparing results from the F + A, F + A + P, F + A + R, or F + A + P + R to ISA206 groups. Data are shown as the mean ± standard error of 12 mice per group.

R848 and poly(I:C) as adjuvant reportedly induce a substantial increase in both Th1- and Th2-type immune responses [19,20]. To determine the potential functions of R848 and poly(I:C) accompanied with FMDV antigens in Th1/Th2-type immune response, cytokine levels were assayed. As shown in Figure 3, R848 and poly(I:C) promoted IFN-γ production. In the F + A + R + P group, the level of IFN-γ was fivefold higher than that in the F + A group and a little lower than that in the F + 206 group (*p* > 0.05). However, no significant difference was observed in IL-4 production between the F + A + R + P and F + 206 groups (*p* > 0.05), indicating that R848 and poly(I:C) biased the immune response toward a Th1-type direction.

### Flow cytometry analysis

**Figure 4:**
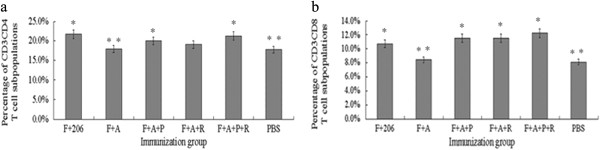
**Effect induced by FMDV with different chemicals on T lymphocyte subsets of mouse spleen.** The proportions of CD3^+^CD4^+^ and CD3^+^CD8^+^ subsets were assessed by two-color flow cytometry analysis. **(a)** CD3^+^CD4^+^ T cells from immunized mouse spleen. **(b)** CD3^+^CD8 ^+^T cells from immunized mouse spleen. One asterisk indicates that *p* < 0.05 when comparing results from the ISA206, F + A, F + A + P, F + A + R, F + A + P + R, or PHA groups to the PBS group. Two asterisks indicate *p* < 0.05 when comparing results from the F + A, F + A + P, F + A + R, or F + A + P + R groups to the ISA206 group. All experiments were obtained in triplicate, and data shown are the mean ± standard error. Representatives from three independent experiments are shown.

To characterize the cell-mediated immune responses, BALB/C mice were sacrificed and a single splenocyte suspension was prepared at 21 dpv. The T cell antigen receptor-CD3 complex plays an important role in the recognition and response to antigens. CD4 and CD8 are T cell surface glycoproteins that recognize major histocompatibility complex (MHC) antigens and play a critical role in signal transduction and T cell activation [21]. When CD3^+^CD4^+^ cells were evaluated post-vaccination, the F + A group remained unchanged relative to the PBS group, and the other groups had higher percentages of this cells than the PBS group. However, no significant difference was observed between the F + 206 and F + A + P + R groups (*p* > 0.05) (Figure 4a). The percentage of CD3^+^CD8^+^ T cells was also evaluated, except for the F + A group, relative to the PBS group. However, when using the same specific antigens, the ability to stimulate CD8 T cell proliferation through different immune formulation was better in the F + A + R, F + A + P, and especially in the F + A + R + P groups (Figure 4b).

## Discussion

The important finding of this study was that R848, poly(I:C), and Al(OH)_3_ enhanced the immune response against the FMDV, which has not yet been reported. The immunization of FMDV formulated with R848, poly(I:C), and Al(OH)_3_ markedly augmented antibody and cellular immune responses. However, weak humoral and cellular responses were elicited by FMDV and Al(OH)_3_ immune formulation.

The immunomodulatory effects of imidazoquinolines have been extensively studied both in mice and humans. Imiquimod, the first synthetic imidazoquinoline, is approved by the FDA for the topical treatment of viruses, such as human papillomavirus and genital HSV-2. R848, another imidazoquinoline, has been shown to preferentially enhance Th1 immune response through the recognition of TLR7 in antigen-presenting cells in mice, especially DCs [22]. Their binding induced the activation of DCs and the production of Th1-biased cytokines, such as TNF-α, IL-12, and IFN-γ. Immune response against FMDV in natural hosts is probably T-cell dependent, and cell-mediated immunity may be involved in the clearance of persistent FMDV [23]. Ideally, a FMDV vaccine should be able to prime Th1/Th2-balanced immune responses to eliminate FMDV infection. The Th1 cytokines induced in vaccinated animals were correlated with the ability to control viral replication. The expression of IL-12 and IFN-γ in immune cells responding to the FMDV reflects a dominance of Th1 type cells that can contribute to virus clearance. In general, Th1 cells are better endowed than Th2 to deal with viral infections [24].

Poly(I:C) could increase Th1 cytokines and type I IFNs by activating innate immunity through TLR3 and some other molecules mediating signal transduction. Then, DC maturation and B cell activation are induced [25]. Potent CD4^+^ T cell and humoral immune responses can thus be induced. Moreover, both R848 and poly(I:C) could generate CD8^+^ T cell immune response through different mechanisms [26,27]. Collectively, these data strongly support the ability of R848 and poly(I:C) as immunomodulators for improving humoral and cellular immune responses.

Pure FMDV protein antigens have poor immunogenicity, which is the main cause of low immune efficacy in FMDV control and prevention. Using adjuvants can improve immunotherapy or vaccines. However, adjuvants used in FMD vaccines are salts of aluminum and mineral oil. Salts of aluminum have good safety record but produce poor T cell responses, and mineral oil produces high Th1 cell response but causes severe local toxicity. As part of the present study, the co-delivery of these materials accompanying FMDV must not induce severe local and general toxicity. Fortunately, observations of mice body weight, diurnal thermal variation of body temperature, animal behaviors, and pathological changes at the injection sites revealed that these materials were completely safe for use (data not shown).

Adjuvants such as alum or ISA206 alone can promote strong antibody responses. However, when added with R848 and poly(I:C), the antibody titers induced by FMDV were even higher than that in the F + A or F + 206 groups at 14 dpv, which indicated that R848 and poly(I:C) may be important for humoral response regulation.

In vitro analyses of the effects of R848 and poly(I:C) on immune cells showed that they induced the up-regulation of IFN-γ production and down-regulation of IL-4, which meant that R848 and poly(I:C) shifted the immune responses caused by FMDV and Al(OH)_3_ toward a Th1 direction. In vivo, R848 and poly(I:C) markedly affected the subpopulation of T lymphocyte cells, which enhanced both the percentages of CD4^+^ and CD8^+^ T cells when the two TLR agonists were administered together. Therefore, the two immunoregulatory materials could work together to improve the cellular responses induced by FMDV antigens formulated with alum.

## Conclusions

The study provided substantial evidence of the use of R848 and poly(I:C) as TRL agonists for FMDV alum vaccines in a mice model. Particular application was demonstrated for inducing a high amount of IgG antibody and Th1-biased immune responses. The findings can serve as a foundation for further studies on these TLR agonists for FMDV protein vaccination in large animals.

## Competing interests

The authors declare that they have no competing interests.

## Authors’ contributions

DL and ZL: conceived and designed the study. CZ, SQ, ZL, PS, YC, YC, HB, YF, PL, XB, and BX performed the data collection. CZ wrote the first draft of the paper, and DL critically revised the manuscript for writing errors and important intellectual content. ZL is the guarantor. All authors approved the final version of the manuscript to be published.
